# Rodent Social Behavior Recognition Using a Global Context-Aware Vision Transformer Network

**DOI:** 10.3390/ai6100264

**Published:** 2025-10-08

**Authors:** Muhammad Imran Sharif, Doina Caragea, Ahmed Iqbal

**Affiliations:** 1 Department of Computer Science, Kansas State University, Manhattan, KS 66506, USA; 2 Division of Information and Computing Technology, College of Science and Engineering, Hamad Bin Khalifa University, Qatar Foundation, Doha P.O. Box 34110, Qatar

**Keywords:** rodent social behavior, behavior recognition, vision transformer (ViT), global context-aware network, deep learning, RatSI dataset

## Abstract

Animal behavior recognition is an important research area that provides insights into areas such as neural functions, gene mutations, and drug efficacy, among others. The manual coding of behaviors based on video recordings is labor-intensive and prone to inconsistencies and human error. Machine learning approaches have been used to automate the analysis of animal behavior with promising results. Our work builds on existing developments in animal behavior analysis and state-of-the-art approaches in computer vision to identify rodent social behaviors. Specifically, our proposed approach, called Vision Transformer for Rat Social Interactions (ViT-RSI), leverages the existing Global Context Vision Transformer (GC-ViT) architecture to identify rat social interactions. Experimental results using five behaviors of the publicly available Rat Social Interaction (RatSI) dataset show that the ViT-RatSI approach can accurately identify rat social interaction behaviors. When compared with prior results from the literature, the ViT-RatSI approach achieves best results for four out of five behaviors, specifically for the “Approaching”, “Following”, “Moving away”, and “Solitary” behaviors, with F1 scores of 0.81, 0.81, 0.86, and 0.94, respectively.

## 1. Introduction

Animal behavior recognition based on video data is an essential task in many scientific areas, such as biology, psychology, and medicine discovery research [1]. In particular, the analysis of rodent behavior is of great interest to researchers in behavioral neuroscience. Due to physiological and behavioral similarities between rodents and humans, researchers can study rodent behaviors to understand human behavior, diseases and response to various treatments, and social interactions, among other things [2,3]. Traditionally, researchers have analyzed the behaviors of rodents in video data manually. This is a time-consuming task and subject to human error and inconsistencies [4]. With the advancement of artificial intelligence and deep learning methods, behavior recognition has been automated through video analysis. Such approaches offer promising results, are highly scalable, and can be used in real time [5]. While recent studies have produced consistently better results for automated behavior recognition [6], there is still significant room for improvement in terms of identifying and classifying individual behaviors, such as grooming or feeding, or social behaviors, such as facing each other or following each other. One of the main challenges in automated approaches is dealing with diverse rat breeds, backgrounds, varying lighting conditions, and overlapping behaviors in video recordings. These factors can significantly impact the accuracy and robustness of behavior recognition models. Additionally, training highly effective deep learning models requires large, well-annotated datasets, but the process of data collection and annotation is both labor-intensive and complex [7].

Early automated methods were based on handcrafted features, like Scale-Invariant Feature Transform (SIFT) and Histogram of Oriented Gradients (HOG) [8], which can take significant human effort and face difficulties when applied to complex patterns. The emergence of Convolutional Neural Networks (CNNs) transformed the field by enabling efficient feature extraction from images and videos. However, CNNs are primarily designed for capturing spatial features and lack the ability to model long-range dependencies, which is critical for recognizing behaviors in sequential video frames [9]. To address this, recurrent architectures such as Recurrent Neural Networks (RNNs) and Long Short-Term Memory (LSTM) networks were introduced, but they often suffer from computational inefficiency [10].

More recently, Transformer models have brought new capabilities to modelling local and global relations, which has modified fundamental deep-learning operational concepts through self-attention [11]. Vision Transformers (ViTs) have the ability to capture long-range dependencies by using the patches of images in a sequence and self-attention mechanism [12,13]. However, standard ViTs often ignore capturing the local features and demand high computational resources [14,15]. Later, hybrid architectures such as Swin and Focal Transformers were proposed to address these challenges. The Swin Transformer incorporates a local feature extraction mechanism using hierarchical shifted windows, while the Focal Transformer balances global context modeling and computational efficiency [16].

Despite these improvements, there remains a gap in the effective capture of global dependencies in a computationally scalable manner. To address this gap, Hatamizadeh et al. [17] introduced the GC-ViT network, which exhibits a hierarchical architecture consisting of global and local self-attention modules. Global query tokens are computed at each stage using modified Fused-MBConv blocks [18]. These are advanced fused inverted residual blocks designed to capture and integrate global context information from different parts of the image. While the short-range information is captured within the local self-attention modules and across all global self-attention modules, query tokens are consistently employed to exchange information with local key and value representations.

In this study, we leverage the GC-ViT network to design an approach for identifying rat social interactions, called Vision Transformer for Rat Social Interactions, or ViT-RSI. Our working hypothesis is that this approach, which captures both local features specific to each rat in an image and also incorporates global features that represent the global image-level interactions between rats, has great potential in accurately identifying social rat behaviors. Along these lines, the main contributions of our work can be summarized as follows:

- We focus on the task of identifying rodent social behaviors and propose an approach that leverages the GC-ViT architecture [17]. We refer to our approach as ViT-RSI.
- This architecture enhances feature representation by integrating multiscale depthwise separable convolutions within a mixed-scale feedforward network and fused MBConv blocks. Using residual connections and depthwise separable operations, this architecture effectively captures rich multiscale contextual information while significantly reducing the computational cost.
- We used the RatSI dataset to experiment with the proposed ViT-RSI model. Specifically, we focused on five behaviors from the RatSI dataset, including “Approaching”, “Following”, “Social Nose Contact”, “Moving away”, and “Solitary”, and showed that our approach outperformed a prior GMM baseline model for four out of five behaviors.

## 2. Related Work

Various machine learning- and deep learning-based methods have been proposed to accurately identify rodent behaviors in video data. We review some single-rat behavior recognition works, as well as works on social rat behavior recognition in the following sections.

### 2.1. Single-Rat Behavior Recognition

Single rat behaviors are generally easier to study and are more widely assessed using various tests and automated systems. These behaviors often center around sensory-motor functions, as well as learning and memory functions in response to testing environments [19], e.g., locomotion, rearing, grooming, eating, drinking, novel object recognition, resting, freezing, etc. A variety of deep learning approaches have been used to detect such behaviors from single frames or short clips. For example, Dam et al. [20] introduced a rodent behavior recognition deep learning method based on the multi-fiber network (MF-Net) [21] and data augmentation techniques, including dynamic illumination changes and video cutout. Experimental results on a dataset including 10 behaviors showed that the proposed model achieved an average recall rate of 65%. However, the learning did not transfer well to settings and behaviors not included in the training set. Le and Murari [22] proposed a deep learning approach that combines Long Short Term Memory (LSTM) memory networks with 3D convolutional networks to identify rodent behaviors. Experimental results show that the proposed model can achieve an accuracy of 95%, which is similar to the human accuracy. Dam et al. [23] showed that some rodent behaviors have a hierarchical and composite structure. Using a Recurrent Variational Autoencoder (RNN-VAE) with real data and Transformer models with synthetic time-series data, the authors pointed out several reasons for misclassifications and suggested that the availability of large amounts of labeled data that capture diverse behavior dynamics has the potential to lead to improved rodent recognition models.

Despite advancements, single-rat behavior recognition still faces several challenges. These include high overlap between poses of different classes (e.g., resting and grooming) and high variance between events of the same class. Further difficulties arise from imbalanced training data for rare but important behaviors and variations in event duration. Training data must be diverse and sufficient to prevent issues [23].

### 2.2. Social Rat Behavior Recognition

There is also significant work on the social behavior of rodents such as attacking, mounting, allogrooming, mimicking, social nose contact, and close investigation, among others. Systems like SLEAP [24], DeepLabCut (with multi-animal support) [25], Alpha-Tracker [26], MARS (Mouse Action Recognition System) [27], SIPEC [28], and LabGym [29] are designed to track and identify multiple animals simultaneously in complex environments, a step that can be crucial for analyzing social interactions. Leveraging one of these systems, DeepOF [30] is an open-source tool designed to investigate both individual and social behavioral profiles in mice using DeepLabCut-annotated pose estimation data. DeepOF offers two main workflows: a supervised pipeline that applies rule-based annotators and pre-trained classifiers to detect defined individual and social traits, and an unsupervised pipeline that embeds motion-tracking data into a latent behavioral space to identify differences across experimental conditions without explicit labels.

Other works have focused specifically on models for identifying social rat behaviors. For example, Camilleri et al. [31] provide tools that capture the temporal information of mouse behavior within the home cage environment and the interaction between cagemates. This research also introduces the Activity Labeling Module for automatic behavior classification from video. Zhou et al. [32] proposed a model for complex social interactions between the mice. This model is based on the Cross-Skeleton Interaction Graph Aggregation Network, which addresses challenges in interaction due to ambiguous movement and deformable body shapes, which is helpful to enhance the representation of the social behaviors of mice. Jiang et al. [33] introduced a dynamic discriminative model and multi-view latent attention to analyze social behavior between mice. This research captures the rich information of mice engaged in social interaction with the help of multi-view video recordings. It addresses the challenges of identifying the behavior from various views. Their experimental results on the PDMB and CRM13 datasets demonstrated strong model performance.

Ru et al. [34] introduced the HSTWFormer transformer, which enhances the recognition of rodent behaviors from pose data. This transformer automatically extracts multiscale and cross-spacetime features without any predefined skeleton graphs. This model, STWA spatial–temporal window attention block, captures both long and short-term features to improve performance and also demonstrated an accuracy of 79.3% for interactive behavior and 69.8% for overall behavior on CRIM13 dataset. On the other hand, this model achieved 76.4% accuracy on the CalMS21 dataset. In light of recent advancements and existing limitations, a novel computer vision-based approach is required to improve the recognition of rodent social behavior.

As prior research has shown, identifying the behaviors of multiple rats is challenging due to factors like occlusion within the field of view, the inherent complexity and subtlety of social behaviors, and the variability between different experimental setups. Most current systems still struggle with recognizing complex social behaviors [35]. To help improve the state of the art in recognizing social rat interactions, we aim to explore the use of a powerful transformer-based approach, GC-ViT, which combines global attention with local self-attention. Furthermore, machine learning has also been used in many areas, such as biomedical robotics [36] and medical imaging [37], highlighting its adaptability and versatility beyond behavior recognition.

## 3. Proposed Methods

We propose the use of a Vision Transformer for Rat Social Interaction (ViT-RSI) model to identifying rat behaviors. This model is based on the Global Context Vision Transformer (GC-ViT) [17]. The GC-ViT model contains a sequence of blocks with four main types of layers. Specifically, each block consists of local Multi-Scale Attention (MSA) with a Multi-Layer Perceptron (MLP) layer and Global MSA with MLP layers. We adapt the GC-ViT model by replacing the MLP layers following the local MSA with a Depthwise Separable Convolution-based Mixed-Scale Feedforward Network (DSC-MSFN) [38], to enhance feature extraction through the cross-resolution property of the DSC-MSFN network. MLPs do not capture the spatial relationships between neighboring regions of the image and also process the image tokens independently [39]. The DSC-MSFN enables the network to learn spatial features at multiple scales, allowing the model to recognize complex behavioral patterns that require both the local and global context of an image [40]. We believe this is useful for recognizing social rat behaviors, where we need to capture local rat features and also global image-level features when the rats occur in different regions of the image.

**Figure 1.**
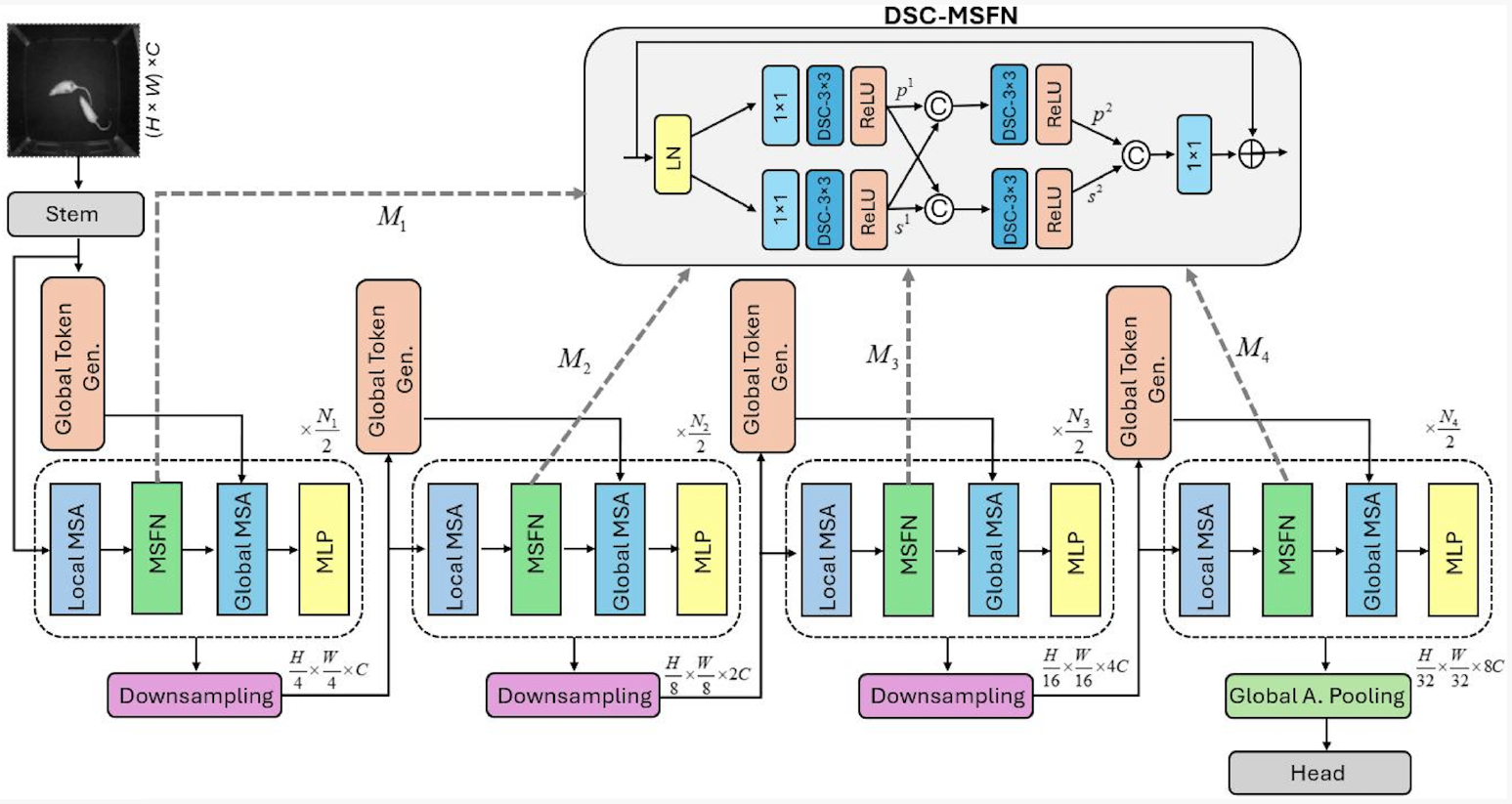
The proposed method architecture is based on multiple stages. During each stage, the query generator handles global query tokenization, extracting long-range dependencies through interactions with local keys and values. The local and global context selfattention layers are used.

The architecture is illustrated in Figure 1. The model takes an input image with resolution $x\in {\mathbb{R}}^{H\times W\times C}$, where overlap patches are obtained using 3 × 3 convolution operations with a stride rate of 2 and the same padding. These patches are mapped into a C-dimensional embedding space using another set of 3 × 3 convolution operations with the same stride and padding. The flow of the proposed method is based on four consecutive blocks, each including Local MSA, DSC-MSFN, global MSA, and MLP. Each block’s downsampling is also connected to the global token generation module and the next similar consecutive block.

Each architecture stage combines local self-attention for capturing fine-grained features and global self-attention using global tokens. The DSC-MSFN replaces standard MLPs to extract spatial features at multiple scales, and downsampling modules reduce dimensionality while preserving necessary information. The final global pooling aggregates features for classification.

## 4. Experimental Setup

### 4.1. Dataset

This research employed the RatSI (Rat Social Interaction) dataset [41] to evaluate the performance of the proposed model in recognizing rat behaviors. This dataset included 9 videos recorded within a 90 × 90 cm PhenoTyper observation cage. Each video included two rats which are interacting with each other without the presence of additional objects in the cage. Each video is 15 min long and captured at 30 fps, nearly 2600 frames per minute. Each frame is labeled by experts.

**Figure 2.**
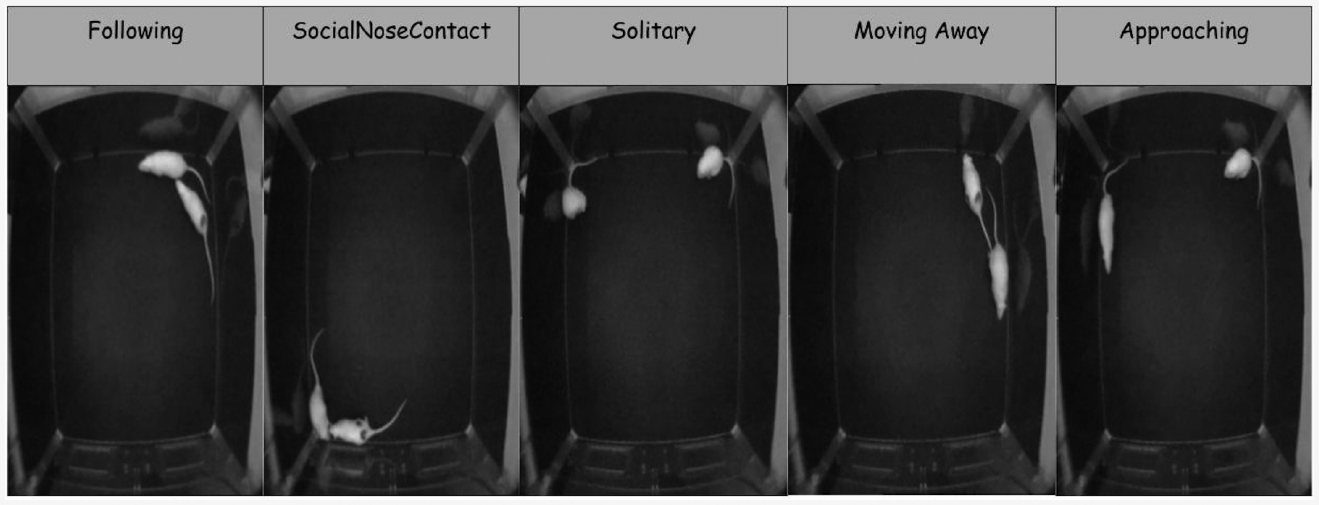
Example frames from the RatSI dataset illustrating the five annotated behaviors: Following, Social Nose Contact, Solitary, Moving Away, and Approaching. Each frame is extracted from video annotations provided in the dataset. These examples represent the visual appearance of each behavior class used for model training and evaluation.

Similarly to [41], in this research, we focus on five specific behaviors out of ten, as the other behaviors are not well-represented in the dataset. The behaviors used are “Solitary”, “Moving Away”, “Following”, “Social nose contact”, and “Approaching”. These behaviors are particularly challenging to analyze due to their similarities. The descriptions of the behaviors are provided in Table 1. Furthermore, sample frames illustrating each of the behaviors of interest are shown in Figure 2. Statistics about the dataset, including the number of frames for each behavior in each video are shown in Table 2. This table also shows how the videos were split into train/test sets in our experiments. Validation frames were sampled from the training videos.

### 4.2. Evaluation Metrics

The performance of the proposed model is evaluated using four key metrics: precision (PRE), recall (REC), F1 score (F1), and accuracy (ACC). Precision measures the positive predictive value. Recall, known as sensitivity, reflects the true positive rate, which means how many frame behaviors are accurately recognized by the proposed model. The F1 score represents the tradeoff between precision and recall, defined as the harmonic mean of precision and recall. Lastly, accuracy provides the overall performance over all classes.

### 4.3. Training Details

We experimented with several variants of the ViT-RSI model, including the tiny, small, and base variants. The specific hyperparameters used for these variants are shown in Table 3. Each model is trained over 50 epochs, with 7 videos used for training and validation, and the remaining 2 videos used for testing. The RatSI dataset provides one annotation file per video with frame-by-frame behavioral labels. We extracted frames according to these annotations, assigned labels directly from the provided files, and split them by video ID to ensure no cross-video leakage between train, validation, and test subsets. The input frames are resized to 224 × 224, and the pixel values are normalized to the range [0, 1] by dividing each pixel value by 255. No additional data augmentation was applied in this study. The model is trained on a GPU A100 system using the TensorFlow framework. Each epoch required approximately 89.6 min, resulting in a total training time of about 74 h and 40 min. Peak GPU memory usage during training was approximately 28 GB (measured with nvidia-smi). At inference, the model was able to process frames in real time (batch size = 8; input size = 224 × 224) on a single NVIDIA A100 GPU, demonstrating high computational efficiency. The initial learning rate was set at 0.001. ReduceLROnPlateau was utilized for early stopping regulation to reduce the learning rate by 0.1 factor when validation loss failed to improve after 20 epochs with the minimum delta of 0.0001. The Stochastic Gradient Descent (SGD) optimizer was used for training the model and weight cross-entropy loss was used to mitigate the issue of imbalances in the dataset during the model training. We also tested Adam in preliminary trials, but it showed faster overfitting and reduced validation performance compared to SGD; hence, we report only SGD results in this study. The weights for each class were calculated using the formula $w_{g}=\frac{p_{\text{samples}}}{p_{\text{classes}}\times p_{\text{samples}}}$ where *w*_*g*_ is the weight of the class *g*, *p*_samples_ represents the total number of samples, and *p*_classes_ means the total number of classes. This inverse class weighting scheme enabled the model to learn better features from the minority class with minimum examples. Hyperparameters were chosen empirically based on validation performance in preliminary experiments. Batch size was fixed at 8, dropout rate at 0.2, and the initial learning rate at 0.001. These settings provided stable training and were retained across all experiments. The final chosen values are reported in Table 3.

**Figure 3.**
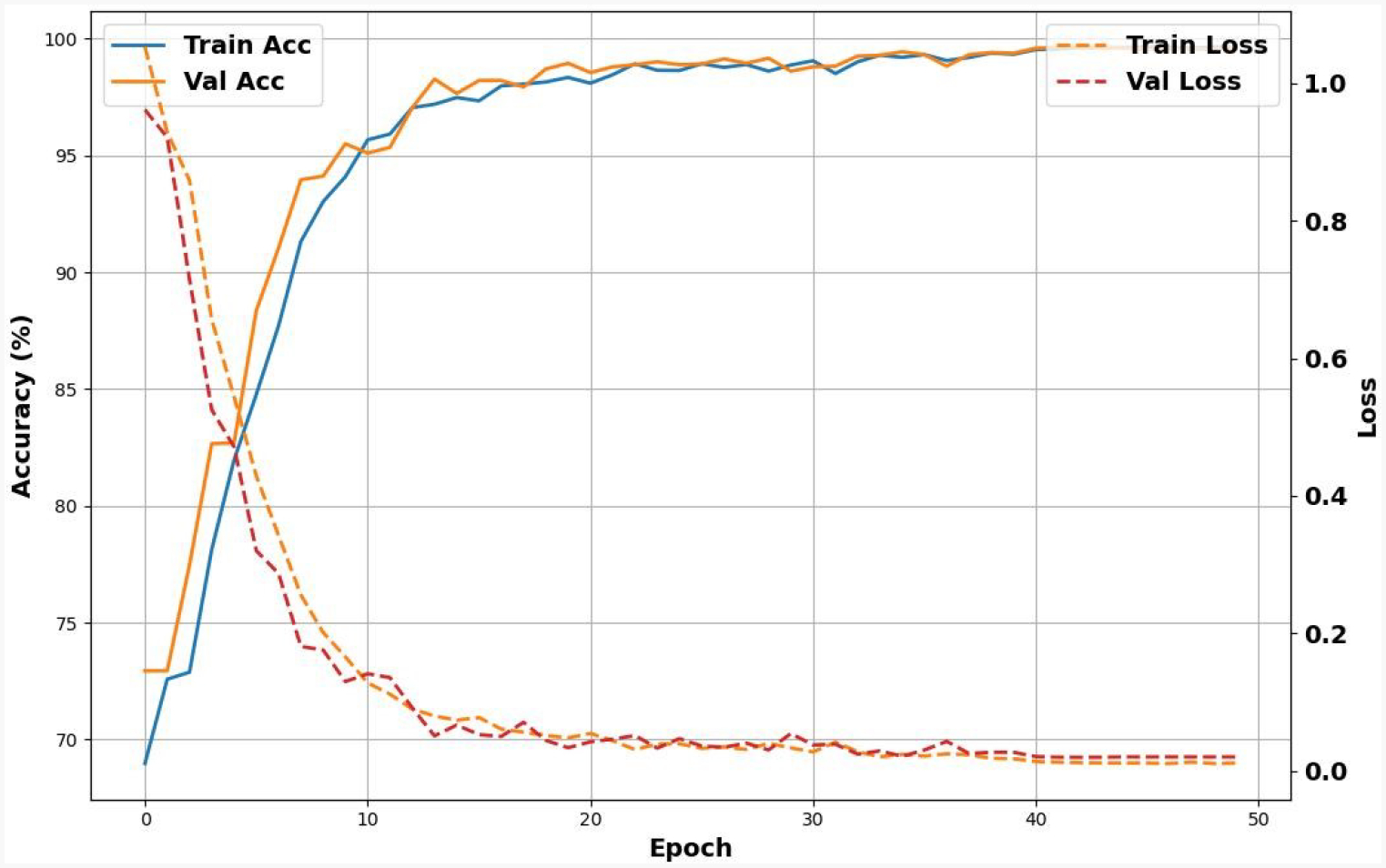
Learning curves showing the validation accuracy and validation loss by comparison with the training accuracy and loss. The curves suggest that the model converges after approximately 50 epochs.

Figure 3 represents the visualization of training and validation accuracy and loss. It shows that the model achieves good results after 10 epochs. However, the model reaches optimal performance at 50 epochs, with maximum training and validation accuracy. The loss decreases to below the 0.5 threshold. This indicates that the model has achieved better convergence over the training period.

#### Workflow Summary

To provide a concise overview of the proposed pipeline, Algorithm 1 summarizes the end-to-end workflow from raw data to final classification.

**Algorithm 1:** End-to-End Workflow of ViT-RSI for Rodent Social Behavior Recognition

1:	**Input:** 9 RatSI videos with corresponding frame-level annotations
2:	Split videos into training/validation (7) and test (2) by video ID (no leakage)
3:	**for** each video **do**
4:	Extract annotated frames and assign labels from annotation files
5:	Resize frames to 224 × 224 and normalize pixel values to [0, 1]
6:	**end for**
7:	Initialize ViT-RSI model (Tiny/Small/Base configuration)
8:	**for** each epoch **do**
9:	Train model with SGD optimizer and weighted cross-entropy loss
10:	Apply ReduceLROnPlateau scheduler (monitor validation accuracy)
11:	Apply early stopping if no improvement is observed
12:	**end for**
13:	**Output:** Trained ViT-RSI model for inference on unseen test videos

### 4.4. Baseline Models and Hyperparameters

We experimented with several variants of the Swin transformer as baselines, including the tiny, small, and base variants to allow for a fair comparison with the similar variants of the ViT-RSI model.

## 5. Results and Discussion

### 5.1. ViT-RSI Versus the Swin Transformer Baseline

Table 4 shows the results of the three variants of the ViT-RSI approach (ViT-RSI-T, ViT-RSI-B, and ViT-RSI-M) by comparison with the corresponding variants of the Swin Transformer baseline variants [42] (specifically, Swin-T-T, Swin-T-S, Swin-T-B). As we can observe, the Swin-T-T achieved an F1 score of 0.62, and an accuracy value of 0.74. For Swin-T-S, the results increased by 2% in the majority of performance measures, while for Swin-T-B the results are better than the results of the tiny model by a 4% increment in most performance measures. Our tiny ViT-RSI-T model achieved an F1 score of 0.72 and an accuracy value of 0.84. The small model, ViT-RSI-S, achieved approximately 2–3% improvement in most performance measures. Finally, the base model, ViT-RSI-B, achieved the best results overall, with an F1 score of 0.78, and an accuracy value of 0.90. It is also worth noting that the precision of the ViT-RSI-B model has a precision value of 0.84, which is slightly higher than the recall with a value of 0.78. But overall, the model performs well in both precision and recall metrics. In terms of model size, as can be seen in the P(M) column of Table 4, the best performing ViT-RSI-B model has a size of 11.7 million parameters, which is significantly smaller than the size of all the Swin Transformer variants. Thus, the ViT-RSI-B model delivers better performance with fewer parameters and computation. We will use this model for the remainder of the experiments in this study.

In this study, we used the Swin Transformer, one of the baselines, because of its tiny, small, and base variations, which provide a fair comparison across different model scales. The Swin Transformer is also widely recognized as a powerful transformer for computer vision tasks, while other transformers are promising but have not been tested on the RatSi dataset, and often need pre-processing for the model training. However, this presents a gap where future research can work to analyse and include these broader benchmarks for more effective evaluation.

### 5.2. Action-Wise Results

Table 5 shows the results of the ViT-RSI-B model separately for each RatSI action/behavior included in our experiments, specifically, “Approaching”, “Social Nose Contact”, “Following”, “Moving Away”, and “Solitary”. The results indicate that the “Solitary” action achieved the best results in all performance measures with a precision value of 0.93, a recall value of 0.95, and an F1 score of 0.94. The “Moving Away” behavior has the second-best results, with an F1 score of 0.86, followed by the “Approaching” and “Following” behaviors, both with an F1 score of 0.81. The “Social Nose Contact” behavior had weaker results compared to the other behaviors, with an F1 score of 0.48.

**Figure 4.**
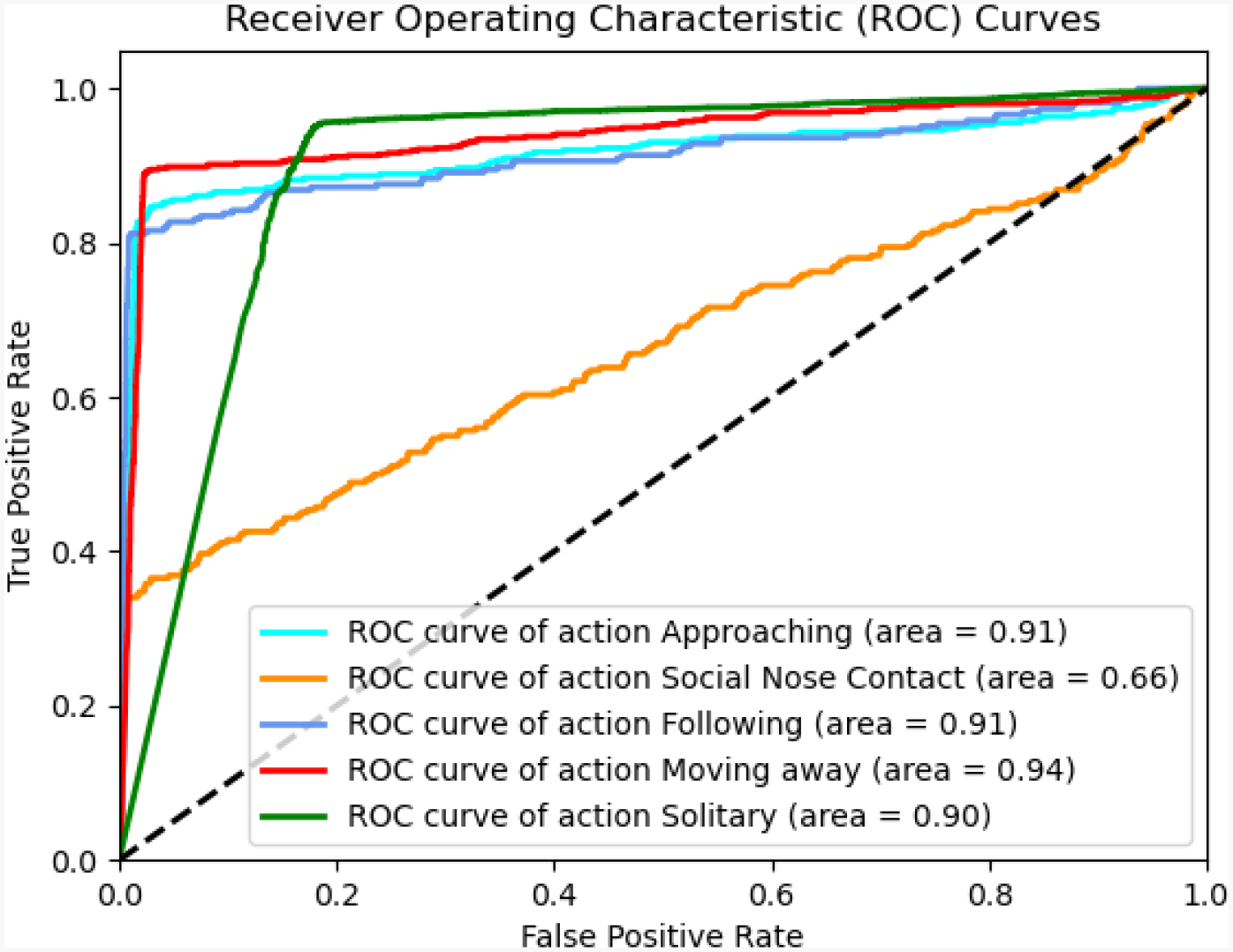
Receiver Operating Characteristic (ROC) curves for the five annotated behaviors in the RatSI dataset: Approaching, Social Nose Contact, Following, Moving Away, and Solitary. The Area Under the Curve (AUC) is reported for each class, with Moving Away achieving the highest AUC of 0.94 and Social Nose Contact the lowest at 0.66. These curves illustrate class-wise performance and highlight variation in discriminative ability across behaviors.

Figure 4 shows the ROC curves, together with the corresponding Area Under the Curve (AUC) values, for the five behaviors in our study, as an alternative way to visually evaluate performance. It is easy to see that the model did not perform well on the “Social Nose Contact” behavior. We can also notice that the “Moving Away” behavior has the highest AUC value of 94, even higher than the “Solitary” behavior, which had the best F1 score.

**Figure 5.**
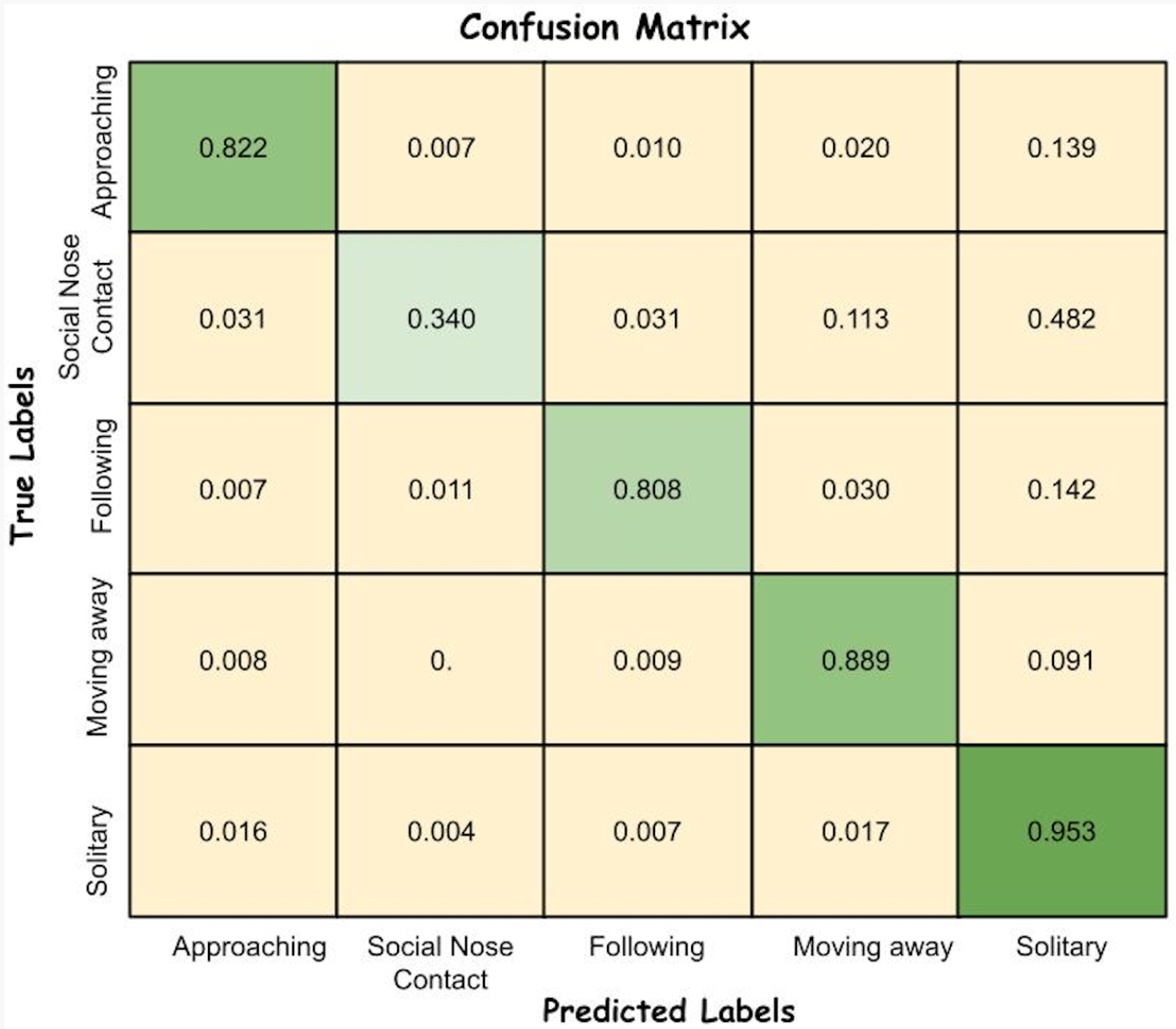
Confusion matrix displaying the classification results.

Figure 5 shows the confusion matrix for the best ViT-RSI-B model, which provides insights into how behaviors are misclassified. From the confusion matrix, we observed that only 34% of “Social Nose Contact” frames were correctly classified, while approximately 48% were misclassified as “Solitary”. Smaller portions were also confused with “Moving Away” (11%) and with “Approaching” or “Following” (about 3% each). For example, the “Social Nose Contact” behavior is generally misclassified as “Solitary” behavior, as the rats are probably not moving much when they are close to each other. Therefore, we can infer that misclassifications may occur due to overlapping behaviors and the way the model prioritizes one over another. Some behaviors are misclassified as “Solitary” more often than other behaviors, which suggests that the model might have some bias towards this behavior, thus explaining the good performance observed obtained by the model on the “Solitary” behavior.

Overall performance indicated strong performance; the results vary across behaviors, but the “Social Nose Contact” showed the weakest performance. This can be because of the limited number of labels for this behavior, which is leading to class imbalance and is particularly compensated for by weight loss. In addition, the brief duration of nose contact events limits the availability of consecutive frames, which reduces the number of representative frames for practical model training. From the single frames, these events are also visually similar to the “Approaching” or “Following” behaviors which often occur immediately before or after nose contact. Since the current framework relies on frame-level classification, it does not capture the temporal transitions that would help differentiate these behaviors. These factors explain the reduced accuracy for “Social Nose Contact” and suggest that future extensions using temporal modeling or targeted augmentation for rare behaviors may improve performance.

### 5.3. Comparison with Results of Prior Work

Table 6 shows a comparison of the action-wise performance of the ViT-RSI model in terms of F1 score by comparison with results for those behaviors from the prior work that introduced the RatSI dataset [41]. While not exactly the same train/test data split was used in the prior work by Lorbach et al. [41], the results suggest that the ViT-RSI model has better performance than the prior model for four out of five behaviors. For example, the ViT-RSI model achieves a high F1 score of 0.81 for both “Approaching” and “Following” behaviors, while the prior model has F1 scores of 0.43 and 0.53 for “Approaching” and “Following”, respectively. For the “Moving Away” behavior, the model achieves an F1 score of 0.86 by comparison to the F1 score of 0.26 of the prior approach. The results are more comparable for the “Solitary” behavior, with an F1 score of 0.94 for ViT-RSI versus an F1 score of 0.80 for the prior approach, suggesting that the “Solitary” behavior might be the easiest to identify among the five behaviors included in our study. These results highlighted the importance and capabilities of the Vision Transformer architecture for accurate behavior recognition.

### 5.4. Ablation Study

To understand the role of each of the components of the ViT-RSI model, specifically the role of the Local MSA, Global MSA, and DSC-MSFN module, we have performed an ablation study, where we compare the baseline model, without any of these components, with variants where we add one component at a time incrementally. The results of the ablation study are shown in Table 7. As can be seen in the table, the baseline model achieves an F1 score of 0.73 and an accuracy value of 0.85. With the addition of the Local MSA, a 1% improvement is observed in most performance measures. Furthermore, when adding the Global MSA block, there is an improvement of approximately 2% in most performance measures. Finally, when we also added the DSC-MSFN module, the model achieved its best performance. This shows that all the components of the ViT-RSI model, including Local MSA, Global MSA, and DSC-MSFN are necessary for best performance.

### 5.5. Error Analysis

**Figure 6.**
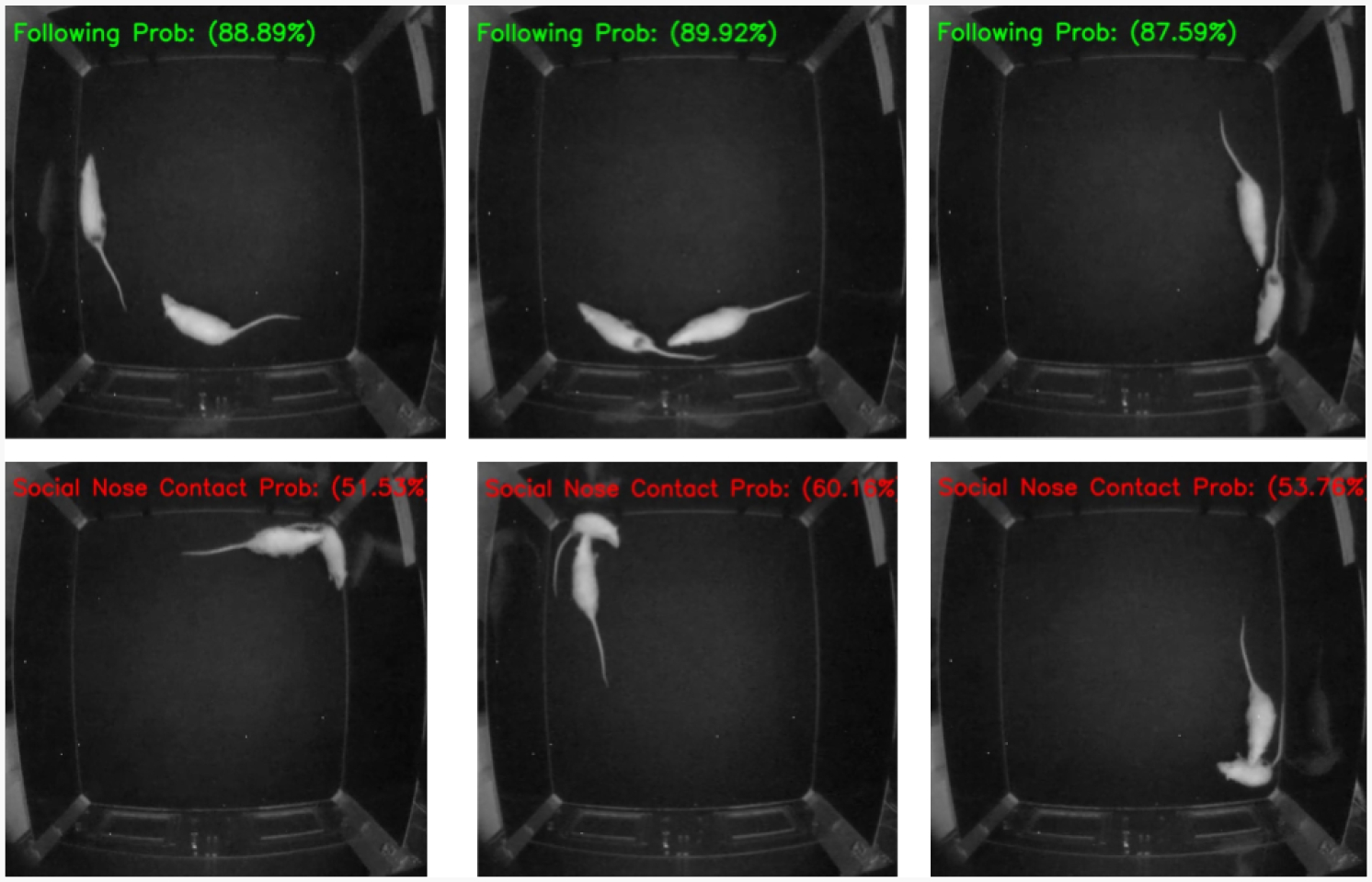
Sample frames from test video demonstrating the performance of the ViT-RSI model in classifying rodent social behaviors.

Figure 6 shows example frames from the test video to illustrate the model’s prediction behavior. While the study includes five behavior classes, this figure focuses on two behaviors: “Following” and “Social Nose Contact”. The top row shows correct “Following” predictions, with high confidence. The bottom row includes “Social Nose Contact” predictions, with relatively low confidence, indicating uncertainty or possible misclassification.

In this analysis, confidence means the probability score noticed by the softmax layer of the ViT-RSI model. For example, the first frame in Figure 6, the label “Following Prob: 88.89%” indicates that the model predicted the “Following” class with 88.89% probability for that frame. High probability means more substantial confidence in the prediction, whereas lower values show more uncertainty and a higher chance of misclassification/error.

## 6. Conclusions

In this paper, the Rodent Social Behavior Recognition Vision Transformer (ViT-RSI) method is proposed, which consists of four blocks having four layers—local MSA, DSC-MSFN, Global MSA, and MLP, in this sequence. The method is evaluated using the RatSI dataset from which five behaviors are selected for experiments—“Approaching”, “Social Nose Contact”, “Following”, “Moving Away”, and “Solitary”. Experimental results show that the ViT-RSI model can accurately predict most of the behaviors considered. However, possibly overlapping behaviors (e.g., frames where the rats may exhibit both the “Solitary” and “Social Nose Contact” behaviors) may prevent the model for performing well on some of the less represented behaviors. The ViT-RSI results proved to be superior to the results of the prior model that was tested on this dataset [41] for four out of five behaviors, and also consistenly better than the results of the state-of-the-art Swin Transformer. Furthermore, compared to the Swin Transformer network (which has 28.2 million parameters for the smallest variant), the best and largest ViT-RSI utilizes only 11.7 million parameters. This indicates that our model not only performs well, but is also computationally inexpensive, lightweight, and easily deployable.

## Figures and Tables

**Table 1.** List of RatSI behaviors [41] used in this study, together with their descriptions.

Behavior	Description
**Approaching**	One rat moves closer to the other.
**Following**	One rat walks behind the other.
**Social Nose Contact**	The two rats touch noses or bodies.
**Moving away**	One rat moves away from the other.
**Solitary**	The rats are alone, no interaction.

**Table 2.** Dataset statistics: The number of frames of each behavior in each video is shown, together with the total number of frames for each behavior and globally in the dataset. The split of the videos between train and test subsets is also shown. Validation frames are randomly sampled from the train videos.

Video	Approaching	Following	Moving Away	Nose Contact	Solitary
1 (Train and Validation)	1405	2150	985	1799	12,281
2 (Train and Validation)	1066	593	592	1754	14,709
3 (Train and Validation)	1083	2522	482	1106	15,428
4 (Test)	1528	444	931	2621	14,776
5 (Test)	1310	2955	880	1067	14,297
6 (Train and Validation)	2443	2141	1503	1980	13,152
7 (Train and Validation)	2058	3359	1202	1865	11,405
8 (Train and Validation)	2066	2696	1391	3422	12,323
9 (Train and Validation)	2259	2017	936	4141	11,295
**Train (80%)**	9904	12,382	5672	12,853	72,474
**Validation (20%)**	2476	3095	1418	3213	18,118
**Test**	2838	3399	1811	3688	29,073
**Total**	15,218	18,877	8902	19,755	119,666

**Table 3.** Hyperparameters used in the ViT-RSI tiny, small, and base model configurations, respectively.

Hyperparameter	Tiny	Small	Base
Resolution	224	224	224
Drop Path Rate	0.2	0.3	0.5
Input Channels	3	3	3
No. of Classes	5	5	5
QKV Bias	True	True	True
Depths	[3,4,19,5]	[3,4,19,5]	[3,4,19,5]
Num Heads	[2,4,8,16]	[3,6,12,24]	[4,8,16,32]
Window Size	[7,7,14,7]	[7,7,14,7]	[7,7,14,7]
Dim	64	96	128
MLP Ratio	3	2	2
Layer Scale	-	1 × 10^−5^	1 × 10^−5^

**Table 4.** Performance comparison between variants of ViT-RSI (tiny, small, base) and the corresponding variants of the Swin Transformer baselines. The number of parameters of each model is also shown in the P(M) column (in millions). The best performance results are highlighted in **bold** font.

Method	P(M)	PRE	REC	F1	ACC
Swin-T-T	28.2	0.68	0.60	0.62	0.74
Swin-T-S	49.4	0.70	0.62	0.64	0.77
Swin-T-B	88.6	0.72	0.64	0.66	0.78
ViT-RSI-T	10.0	0.78	0.68	0.72	0.84
ViT-RSI-S	10.1	0.81	0.70	0.75	0.87
ViT-RSI-B	11.7	**0.84**	**0.76**	**0.78**	**0.90**

**Table 5.** Action-wise results on RaTSI dataset.

Action	PRE	REC	F1 Score
Approaching	0.80	0.82	0.81
Social Nose Contact	0.80	0.34	0.48
Following	0.82	0.81	0.81
Moving Away	0.83	0.89	0.86
Solitary	0.93	0.95	0.94

**Table 6.** Comparative analysis of F1 scores from the ViT-RSI model and Lorbach et al. [41] on five RatSI Behaviors.

Study	Models	Data Split	F1 Scores (Behaviors)
Lorbach et al. [41]	GMM, Expectation Maximization	Divided into three parts: two used for training, one for testing	Approaching: 0.43 Contact: **0.58** Following: 0.53 Moving away: 0.26 Solitary: 0.80
ViT-RSI	Vision Transformer (ViT)	Seven videos for training and validation (80% training, 20% validation), two videos for testing	Approaching: **0.81** Contact: 0.48 Following: **0.81** Moving away: **0.86** Solitary: **0.94**

**Table 7.** Ablation study results. The baseline model does not include any of the Local MSA, Global MSA, and DSC-MSFN components. At each line, another component is added incrementally.

Module	PRE	REC	F1	ACC
Baseline	0.79	0.71	0.73	0.85
+ Local MSA	0.80	0.72	0.74	0.87
+ Global MSA	0.82	0.74	0.76	0.88
+ DSC-MSFN	0.84	0.76	0.78	0.90

## Data Availability

This study used publicly available datasets. The Rat Social Interaction dataset (RatSI) is available at https://mlorbach.gitlab.io/datasets/ (accessed on 14 August 2024).
